# Predictors of postoperative complications in elderly patients undergoing pulmonary lobectomy: a retrospective cohort study

**DOI:** 10.3389/fmed.2026.1789608

**Published:** 2026-08-11

**Authors:** Ke Yu, Lijun Hua, Xiaohao Yan, Mei Li

**Affiliations:** 1 Health Management Center, Sichuan Clinical Research Center for Cancer, Sichuan Cancer Hospital & Institute, Sichuan Cancer Center, Affiliated Cancer Hospital of University of Electronic Science and Technology of China, Chengdu, China; 2 Jingtang First People’s Hospital, Jingtang, China; 3 Chengdu Huake Biology Research Center, Chengdu, China; 4 Department of Abdominal Oncology, Sichuan Clinical Research Center for Cancer, Sichuan Cancer Hospital & Institute, Sichuan Cancer Center, Affiliated Cancer Hospital of University of Electronic Science and Technology of China, Chengdu, China

**Keywords:** age-adjusted Charlson comorbidity index score, elderly patients, non-small cell lung cancer, postoperative complications, pulmonary lobectomy

## Abstract

**Background:**

Elderly patients undergoing pulmonary lobectomy for lung cancer are at increased risk of postoperative morbidity. Identifying modifiable risk factors and establishing a practical risk stratification method are essential for improving perioperative management.

**Methods:**

This retrospective cohort study included 212 consecutive patients aged ≥65 years who underwent pulmonary lobectomy for non-small cell lung cancer from 2020 to 2024. Demographic, clinical, and surgical data were collected. The primary outcome was major postoperative complications (Clavien-Dindo grade ≥III). Independent predictors were identified using multivariable logistic regression, and a simplified risk score was constructed. Model performance was assessed using the area under the receiver operating characteristic curve (AUC) and the Hosmer-Lemeshow goodness-of-fit test.

**Results:**

Major complications occurred in 73 patients (34.4%). Multivariable analysis identified three independent predictors: age ≥70 years (OR = 2.26, 95% CI: 1.18–4.42), Age-Adjusted Charlson Comorbidity Index (ACCI) ≥ 6 (OR = 3.70, 95% CI: 1.95–7.25), and preoperative hemoglobin <12 g/dL (OR = 3.04, 95% CI: 1.32–7.19). The multivariable model showed good discrimination (AUC = 0.712, 95% CI: 0.645–0.789) and calibration (Hosmer-Lemeshow *p* = 0.562). A simple risk score assigning one point per predictor demonstrated comparable discrimination (AUC = 0.699, 95% CI: 0.634–0.738) and a stepwise increase in complication rates: 26.0% (score 1), 54.7% (score 2), and 63.6% (score 3).

**Conclusion:**

A risk score based on age ≥70 years, ACCI ≥6, and preoperative anemia effectively stratifies elderly patients by their risk of major complications after pulmonary lobectomy. This simple tool may facilitate preoperative counseling, guide targeted optimization, and inform perioperative resource allocation, though external validation is warranted.

## 1 Introduction

Lung cancer remains highly prevalent among elderly adults, and anatomical lobectomy is still considered the standard radical treatment for patients who meet surgical indications (1, 2). However, elderly patients often have markedly reduced physiological reserves and a higher prevalence of comorbidities (such as cardiovascular and metabolic diseases), which together substantially increase the risk of postoperative complications after lobectomy (3). These complications not only impede early recovery and prolong hospitalization but may also adversely affect long-term survival and quality of life (4). Therefore, accurately identifying high-risk individuals and implementing individualized perioperative management strategies are critical for improving surgical outcomes in this population. Age-related physiological decline may reduce tolerance to thoracic surgery in elderly patients. Reduced respiratory reserve, impaired mucociliary clearance, and diminished cardiometabolic resilience may increase susceptibility to postoperative pulmonary complications and delay recovery after pulmonary lobectomy.

Current evidence has established prolonged operative time as an independent predictor of complications after lobectomy (5). In patients undergoing video-assisted thoracoscopic surgery (VATS), factors such as comorbidity burden, male sex, and intraoperative blood transfusion have also been linked to increased postoperative morbidity (4, 6–8). Among the factors influencing postoperative pulmonary complications, smoking is widely considered an independent risk factor (9). At the same time, chronic obstructive pulmonary disease (COPD) is also considered a key factor increasing the risk of complications (10). It should be noted, however, that most of these studies were conducted in general adult populations, while focused analyses on elderly patients—a distinct high-risk subgroup—remain limited. More recently, geriatric-specific conditions such as frailty, nutritional status, and sarcopenia have gained increasing attention in perioperative risk assessment. Evidence from various surgical fields suggests that these factors are closely linked to adverse postoperative outcomes, yet their specific impact on elderly patients undergoing pulmonary lobectomy remains poorly understood. Similarly, the Age-Adjusted Charlson Comorbidity Index (ACCI) has seen growing use in thoracic surgery as a comprehensive tool for evaluating comorbidity burden, but its role in elderly lobectomy patients has not been systematically examined.

Despite the availability of various risk assessment tools, several challenges limit their routine clinical application in elderly surgical patients. Comprehensive geriatric assessments, while informative, are often time-consuming and require multidisciplinary input, making them difficult to implement in busy preoperative settings. Other existing prediction models may rely on variables that are not routinely collected or require complex calculations, hindering their utility for rapid bedside risk stratification. Consequently, there remains a need for a simple, practical tool that can efficiently identify high-risk elderly patients using readily available clinical parameters. Early identification and modification of potentially reversible risk factors—including anemia, malnutrition, impaired pulmonary function, frailty, and sarcopenia—are essential for preoperative optimization in elderly patients (11). Nevertheless, robust evidence directly linking these factors to postoperative complications in this specific population is still insufficient. To address this gap, we conducted a retrospective cohort study aimed at identifying independent predictors of major complications after pulmonary lobectomy in elderly patients, with the ultimate goal of informing improved risk stratification and personalized preoperative management strategies.

## 2 Methods

### 2.1 Study design and participants

This retrospective cohort study enrolled patients aged ≥65 years who underwent pulmonary lobectomy for non-small cell lung cancer at the Affiliated Cancer Hospital of the University of Electronic Science and Technology of China between 1st January 2020 and 1^st^ December 2024. The inclusion criteria were: (1) age ≥65 years, (2) histologically confirmed non-small cell lung cancer, (3) anatomical pulmonary lobectomy (performed via VATS or open thoracotomy), and (4) complete perioperative data. Exclusion criteria were: (1) pneumonectomy or sublobar resection, (2) emergency surgery, (3) concurrent extrapulmonary malignancy, and (4) loss to follow-up within 30 days post-surgery.

This study was conducted in accordance with the Declaration of Helsinki (as revised in 2013) and was approved by the Institutional Review Board of Affiliated Cancer Hospital of the University of Electronic Science and Technology of China (Approval No. 2025–0322). Due to the retrospective nature of the study and the use of anonymized data, the requirement for written informed consent was waived.

### 2.2 Data collection and variables

Data were extracted from institutional electronic medical records using a standardized case report form. Variables collected included: demographic characteristics [age, sex, body mass index (BMI), smoking history], comorbidities [Age-Adjusted Charlson Comorbidity Index (ACCI), American Society of Anesthesiologists (ASA) physical status, COPD, coronary artery disease, diabetes mellitus], preoperative functional status [forced expiratory volume in 1 s (FEV1) percent predicted, diffusing capacity for carbon monoxide (DLCO) percent predicted, serum albumin, hemoglobin], tumor characteristics (size, clinical stage by 8th edition TNM classification, histology), surgical details (surgical approach, operation time, intraoperative blood loss), and postoperative outcomes. The Age-Adjusted Charlson Comorbidity Index (ACCI) was used to quantify baseline comorbidity burden. The ACCI incorporates weighted chronic comorbid conditions together with age adjustment, thereby providing a composite estimate of overall health status and reduced physiological reserve in elderly patients. In the present study, ACCI was analyzed as a clinically practical summary measure of multimorbidity. Clinically relevant thresholds were used to define binary variables: age ≥70 years, ACCI score ≥6, ASA class III/IV (vs. II), hemoglobin <12 g/dL, intraoperative blood loss ≥200 mL, operation time >180 min, FEV1 < 80% predicted, DLCO <80% predicted, albumin <35 g/L, and open thoracotomy (vs. VATS). For variables with occasional missing values (<5%), complete case analysis was applied; cases missing any of the key predictors or the primary outcome were excluded upfront as per the inclusion criteria.

### 2.3 Study outcomes

The primary outcome was major postoperative complications within 30 days of surgery, defined as complications of Clavien-Dindo grade III or higher. According to the Clavien-Dindo classification, grade III complications require surgical, endoscopic, or radiological intervention, grade IV complications are life-threatening and require intensive care management, and grade V represents death. This classification was used to ensure a standardized and clinically meaningful assessment of postoperative morbidity severity (12, 13). Secondary outcomes included pulmonary complications (pneumonia, respiratory failure, prolonged air leak >5 days, or atelectasis requiring bronchoscopy), length of hospital stay, intensive care unit (ICU) admission, 30-day readmission, and 30-day mortality.

### 2.4 Statistical analysis

Continuous variables were presented as mean (SD) or median (IQR), depending on their distribution, and categorical variables as numbers (percentages). For continuous variables, optimal cutoffs for risk stratification were determined using receiver operating characteristic (ROC) curve analysis, selecting the threshold that maximized the Youden index (sensitivity + specificity −1). Variables with *p* < 0.05 in univariate analysis were included in the multivariable logistic regression model. Model selection employed backward stepwise elimination based on the Akaike Information Criterion. The performance of the final multivariable model and the derived risk score was evaluated using ROC curve analysis, with the area under the curve (AUC) calculated to assess discriminative ability. Model calibration was assessed using the Hosmer-Lemeshow goodness-of-fit test, and multicollinearity among independent variables was examined using variance inflation factors (VIF). All analyses were conducted using R software (version 4.5.1; R Foundation for Statistical Computing, Vienna, Austria). A two-tailed *p* < 0.05 was considered statistically significant.

### 2.5 Follow-up and outcome ascertainment

Postoperative outcomes within 30 days of surgery were systematically ascertained through multiple sources. All patients were followed until 30 days post-surgery or death, whichever occurred first. Outcome data were collected via: (1) daily inpatient medical records during the index hospitalization; (2) review of electronic medical records for any readmissions within 30 days; (3) a scheduled outpatient clinic visit at 30 days post-surgery; and (4) telephone interviews for patients who did not attend the scheduled visit. Complete follow-up was achieved for all 212 patients (100% completion rate), as the primary outcome was assessable for every patient through the combination of these methods Table 1.

**Table 1:** Baseline characteristics of study population (*N* = 212).

Variable	Total (*N* = 212)
Age (years), mean ± SD	70.5 ± 4.1
Gender (male), *n* (%)	121 (57.1)
BMI (kg/m^2^), mean ± SD	23.9 ± 3.4
Smoking status, *n* (%)	
Never smoker	53 (25.0)
Ever smoker	159 (75.0)
Smoking history (pack-years), mean ± SD	26.8 ± 28.4
ACCI score, mean ± SD	6.1 ± 2.6
ASA grade, *n* (%)	
II	116 (54.7)
III	91 (42.9)
IV	5 (2.4)
COPD, *n* (%)	75 (35.4)
CAD, *n* (%)	63 (29.7)
Diabetes, *n* (%)	45 (21.2)
FEV1% predicted, mean ± SD	87.4 ± 13.5
DLCO% predicted, mean ± SD	81.8 ± 17.4
Albumin (g/L), mean ± SD	37.2 ± 3.8
Hemoglobin (g/dL), mean ± SD	13.8 ± 1.6
Tumor size (cm), mean ± SD	3.4 ± 2.0
TNM stage, *n* (%)	
IA	109 (51.4)
IB	27 (12.7)
II	56 (26.4)
IIIA	20 (9.4)
Surgical approach, *n* (%)	
VATS	127 (59.9)
Open thoracotomy	85 (40.1)
Operation time (min), mean ± SD	189.9 ± 49.2
Blood loss (ml), mean ± SD	169.5 ± 85.8

BMI, body mass index; ACCI, Age-adjusted Charlson comorbidity index; ASA, American society of anesthesiologists; COPD, chronic obstructive pulmonary disease; CAD, coronary artery disease; FEV1, forced expiratory volume in 1 s; DLCO, diffusing capacity for carbon monoxide; VATS, video-assisted thoracic surgery.

## 3 Results

### 3.1 Clinical characteristics

This retrospective cohort study included 212 elderly patients with a mean age of 70.5 ± 4.1 years; the majority were male (57.1%) and were current or former smokers (75.0%). The population presented with a significant comorbidity burden, evidenced by 51.9% being classified as high-risk (ACCI score ≥6) and 45.3% with ASA grade III/IV. A compromised preoperative status was observed in a considerable proportion, including 35.4% with COPD, 26.9% with albumin levels <35 g/L, and 32.1% with FEV1 < 80% predicted Table 2.

**Table 2:** Univariate regression analysis for major complications.

Characteristics	OR (95% CI)	*p* value
Age (≥70 vs. <70 years)	2.74 (1.44–5.30)	**0.001**
Gender (male vs. female)	0.67 (0.37–1.24)	0.191
BMI (≥24 vs. <24 kg/m^2^)	0.50 (0.25–0.95)	0.123
Smoking history (yes vs. no)	1.02 (0.49–2.18)	0.994
ACCI Score (≥6 vs. <6)	4.21 (2.17–8.32)	**<0.001**
ASA Grade (III/IV vs. II/I)	1.65 (0.90–3.04)	0.110
COPD (yes vs. no)	0.70 (0.36–1.33)	0.291
CAD (yes vs. no)	1.53 (0.79–2.92)	0.206
Diabetes (yes vs. no)	1.36 (0.64–2.81)	0.382
FEV1 (<80% vs. ≥80% predicted)	0.87 (0.45–1.67)	0.757
DLCO (<80% vs. ≥80% predicted)	0.95 (0.51–1.75)	0.884
Hemoglobin (≥12 vs. < 12 g/dL)	2.51 (1.09–5.80)	**0.025**
Albumin (≥35 vs. < 35 g/L)	1.04 (0.52–2.05)	0.912
Tumor size (≥3 vs. <3 cm)	1.10 (0.60–2.02)	0.774
TNM Stage (II/III vs. I)	1.39 (0.67–2.85)	0.385
Surgery (open thoracotomy vs. VATS)	1.27 (0.68–2.34)	0.462
Operation time (>180 vs. ≤ 180 min)	2.42 (1.28–4.65)	**0.004**
Blood loss (≥200 vs. ≤ 200 ml)	1.53 (0.79–2.92)	0.206

BMI, body mass index; ACCI, Age-adjusted Charlson comorbidity index; ASA, American Society of Anesthesiologists; COPD, chronic obstructive pulmonary disease; CAD, coronary artery disease; FEV1, forced expiratory volume in 1 s; DLCO, diffusing capacity for carbon monoxide; VATS, video-assisted thoracic surgery.Bold values denote statistically significant results (*P* < 0.05).

Surgical procedures were predominantly performed via a VATS approach (59.9%), with a median operation time of 189.9 min and 56.1% of surgeries exceeding 180 min. The mean intraoperative blood loss was 169.5 mL, with 29.7% of patients losing ≥200 mL. Tumor characteristics indicated a mean size of 3.4 cm, with the majority being early-stage disease (IA: 51.4%, IB: 12.7%, II: 26.4%, IIIA: 9.4%).

The overall postoperative complication rate was 45.3% (96/212), with major complications (Clavien-Dindo ≥III) occurring in 34.4% (73/212) of patients. Pulmonary complications constituted the most frequent category at 36.3% (77/212), followed by ICU admission (17.0%, 36/212) and 30-day readmission (11.3%, 24/212). Thirty-day mortality was 0.5% (1/212).

### 3.2 Univariate and multivariable analyses

Univariate analysis identified four factors significantly associated with an increased risk of major postoperative complications. Patients with an ACCI score ≥6 had 4.21 times higher odds of complications (95% CI: 2.17–8.32, *p* < 0.001). Age ≥70 years was associated with a 2.74-fold increase in odds (95% CI: 1.44–5.30, *p* = 0.001). An operation time exceeding 180 min increased the odds by 2.42 times (95% CI, 1.28–4.65, *p* = 0.004), and preoperative hemoglobin <12 g/dL was associated with a 2.51-fold increase in odds (95% CI, 1.09–5.80, *p* = 0.025). No statistically significant associations were found for other examined variables, including ASA grade III/IV, surgical approach, intraoperative blood loss, specific comorbidities, or tumor characteristics.

**Figure 1:**
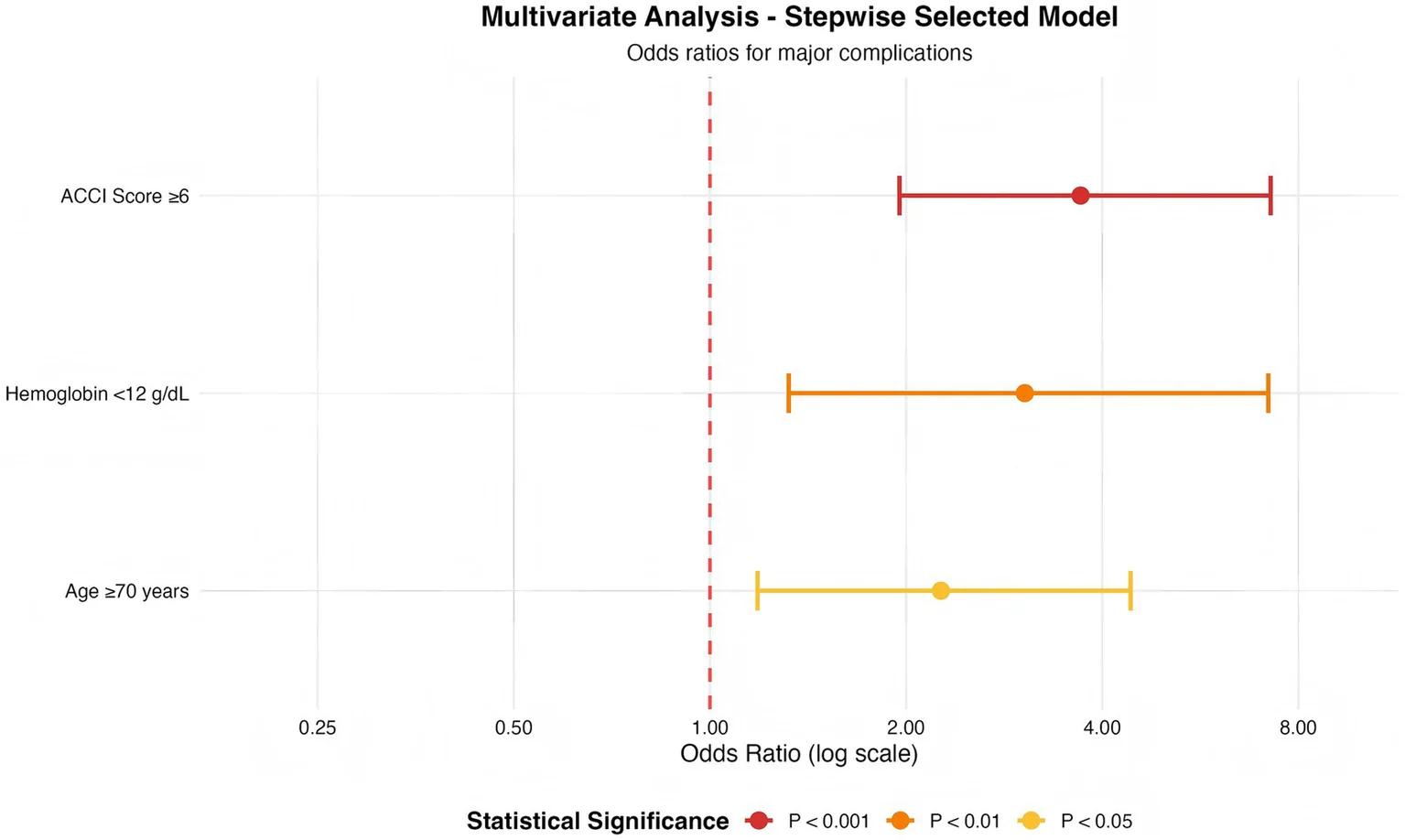
Forest plot of multivariate logistic regression analysis for risk factors of major complications.

Multivariable logistic regression analysis, adjusting for significant univariate predictors, identified three independent predictors of major complications (see Table 3 and Figure 1). An ACCI score ≥6 remained the strongest independent predictor (*β* = 1.308, SE = 0.342, adjusted OR = 3.70, 95% CI: 1.95–7.25, *p* < 0.001). Age ≥70 years (*β* = 0.815, SE = 0.342, OR = 2.26, 95% CI: 1.18–4.42, *p* = 0.015) and preoperative hemoglobin <12 g/dL (*β* = 1.112, SE = 0.439, OR = 3.04, 95% CI: 1.32–7.19, *p* = 0.010) also retained independent significance. However, operation time >180 min, which was significant in univariate analysis, did not remain an independent predictor in the final adjusted model.

**Table 3:** Multivariate regression analysis for major complications.

Characteristics	OR (95% CI)	*p* value
Age (≥70 vs. <70 years)	2.26 (1.18–4.42)	0.015
ACCI score (≥6 vs. <6)	3.70 (1.95–7.25)	<0.001
Hemoglobin (≥12 vs. < 12 g/dL)	3.04 (1.32–7.19)	0.010
Operation time (>180 vs. ≤ 180 min)	1.44 (0.72–2.90)	0.307

ACCI, Age-adjusted charlson comorbidity index.

**Figure 2:**
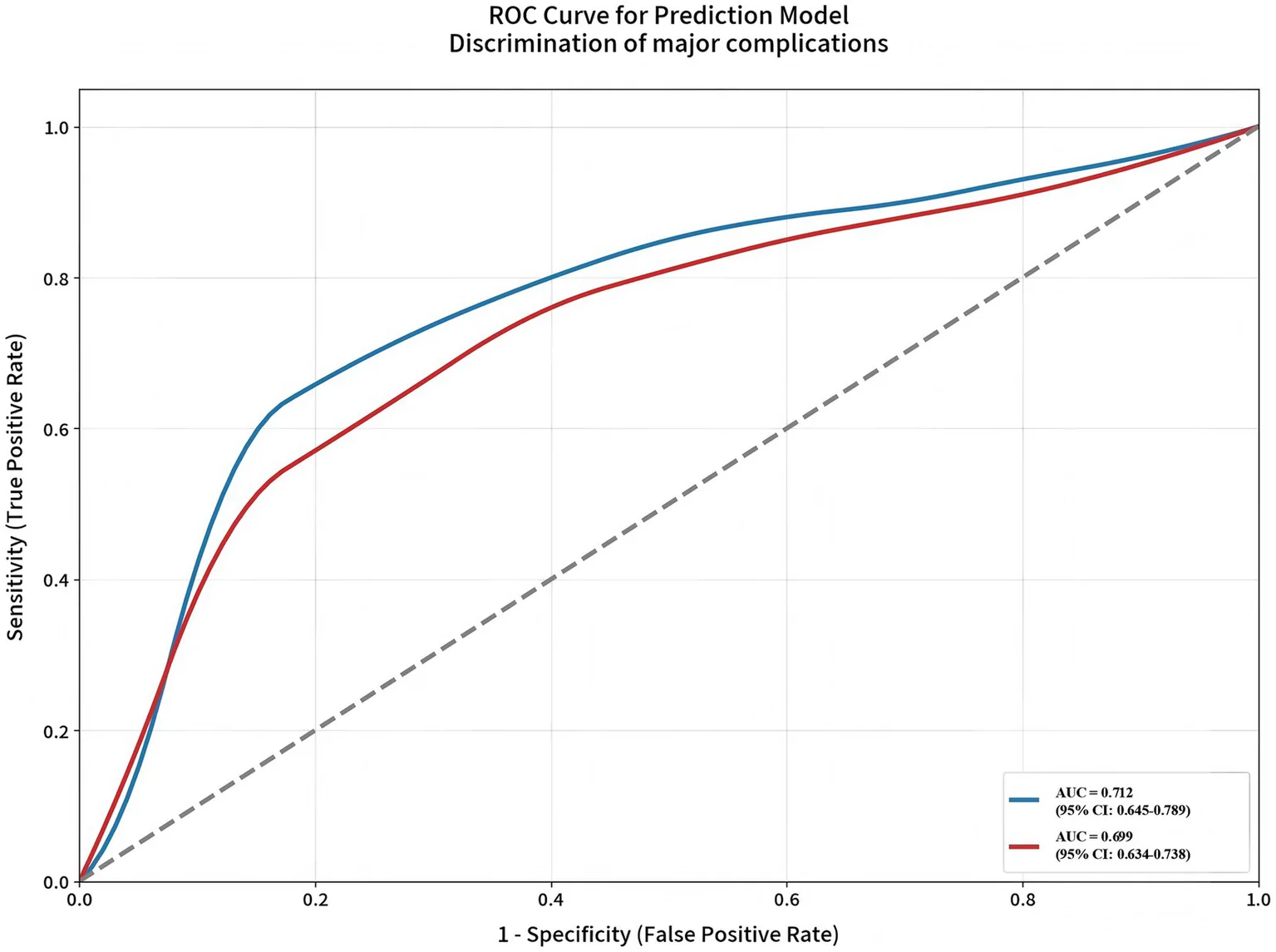
ROC curves for the full multivariable model (blue; AUC = 0.712, 95% CI: 0.645–0.789) and the additive risk score (red; AUC = 0.699, 95% CI: 0.634–0.738).

Model diagnostics were performed to assess the robustness of the multivariable logistic regression. The Hosmer-Lemeshow goodness-of-fit test yielded a *p*-value of 0.562, indicating adequate model calibration. Collinearity diagnostics revealed no substantial multicollinearity among the independent variables, with all variance inflation factors (VIF) below 5 (mean VIF = 3.35). The discriminative ability of the full multivariable logistic regression model, incorporating the regression coefficients for each predictor, was evaluated using ROC curve analysis. The AUC was 0.712 (95% CI: 0.645–0.789), indicating acceptable predictive performance (Figure 2).

### 3.3 Development and performance of the risk score

**Figure 3:**
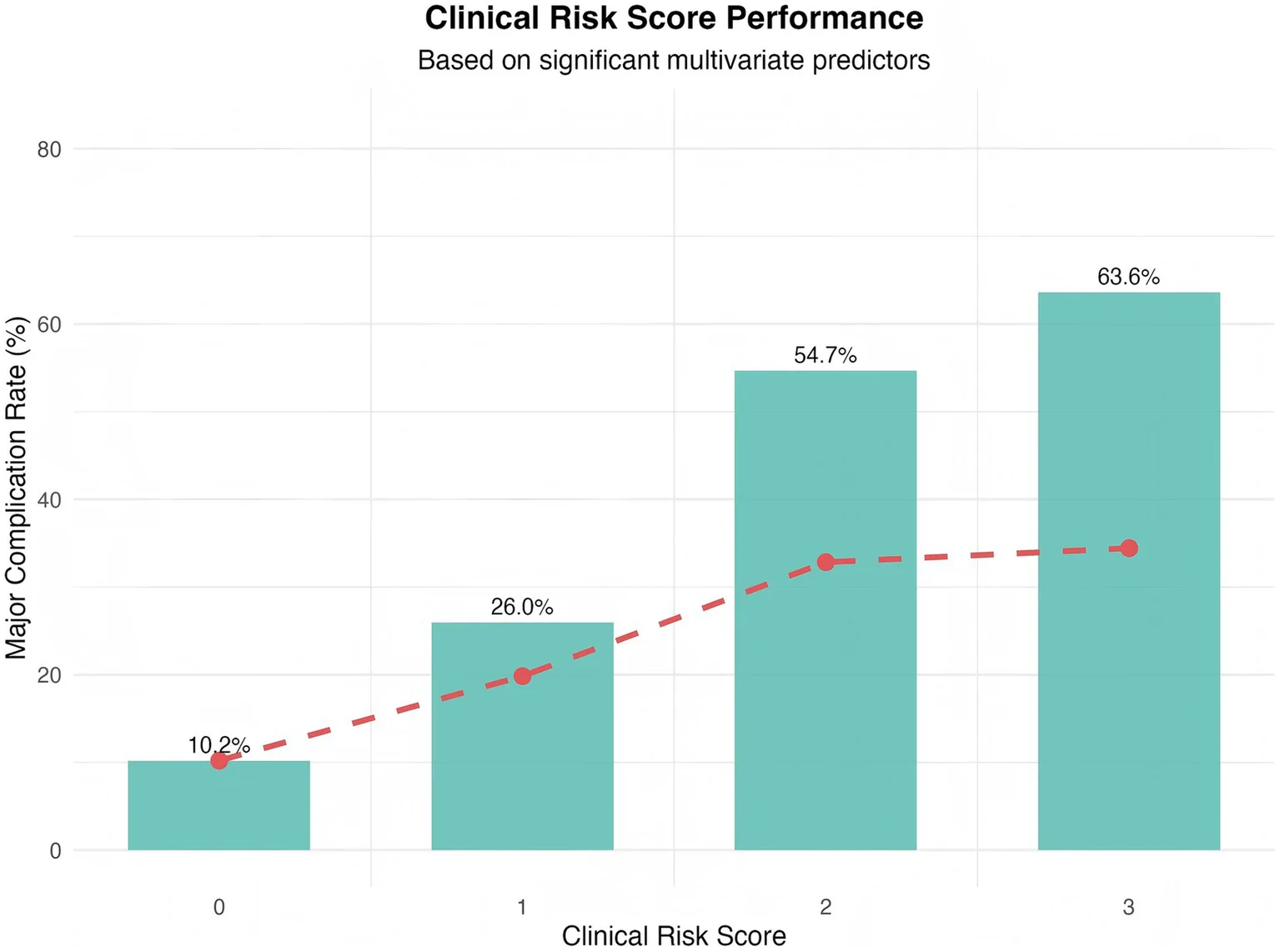
Stepwise increase in major complication rates according to the novel clinical risk score.

A simple clinical risk score was developed by assigning one point for each of the three independent predictors present (ACCI ≥6, age ≥70 years, preoperative Hb < 12 g/dL). The performance of this score is summarized in Figure 3. The major complication rate demonstrated a marked, stepwise increase with higher scores: 26.0% for a score of 1, 54.7% for a score of 2, and 63.6% for a score of 3. The discriminative ability of this simplified score was also evaluated using ROC curve analysis, yielding an AUC of 0.699 (95% CI: 0.634–0.738, see Figure 2), which was comparable to that of the full logistic regression model.

## 4 Discussion

This retrospective cohort study identified three independent preoperative predictors of major complications following lobectomy in elderly patients: age ≥70 years, an ACCI score ≥6, and preoperative anemia (hemoglobin <12 g/dL). By assigning one point for each predictor present, we constructed a simple risk score that effectively stratified patient risk. The incidence of major complications increased markedly with higher scores, from 26.0% for a score of 1, to 54.7% for a score of 2, and 63.6% for a score of 3. This readily applicable score provides a practical tool for preoperative risk assessment and guides individualized perioperative management in this vulnerable population.

An ACCI score ≥6 was associated with an increased risk of complications, confirming the consensus that comorbidity burden is a key factor influencing surgical outcomes. The ACCI serves as a composite metric that quantifies both the number and severity of chronic conditions, thereby reflecting a patient’s overall physiological reserve (14). In elderly patients, a higher ACCI score signifies diminished compensatory capacity to withstand surgical stress, increasing susceptibility to underlying disease exacerbation, postoperative infections, and multi-organ dysfunction. Our findings align with broader surgical literature and provide specific empirical support for its prognostic value in the elderly lung resection population (13, 15–17). The ACCI cutoff of ≥6 used in our study differs from thresholds reported in other studies, and this discrepancy warrants further discussion. Several factors may explain this difference. First, our cohort consisted exclusively of elderly patients (mean age 70.5 years), who inherently have higher comorbidity burdens due to age-related accumulation of chronic conditions. In contrast, studies reporting lower cutoffs often include younger patients, in whom a lower ACCI score may already signify significant comorbidity. Second, our primary outcome focused on major complications (Clavien-Dindo ≥III), which represent a more severe endpoint than all-grade complications used in some studies. A higher threshold may be required to identify patients at risk for more severe outcomes. Third, differences in surgical techniques (e.g., the proportion of VATS procedures) and perioperative management protocols across institutions may also influence the optimal risk threshold. These considerations highlight the importance of population-specific risk stratification and suggest that direct comparisons of cutoff values across studies should be interpreted with caution, taking into account differences in cohort characteristics, outcome definitions, and clinical settings. For patients with ACCI ≥6, targeted preoperative optimization should include comprehensive comorbidity management—such as cardiology consultation for unstable coronary artery disease, endocrinology input for poorly controlled diabetes, and medication reconciliation to withdraw or adjust high-risk perioperative drugs. Individualized anesthesia planning, including careful selection of intraoperative fluids and vasopressors, may also help mitigate risks associated with reduced physiological reserve.

Age ≥70 years emerged as an independent risk factor, aligning with the known trajectory of age-related physiological decline (18). This threshold not only represents chronological age but may also mark a significant inflection point in cardiopulmonary reserve, immune response, and metabolic regulation (19). Collectively, these changes impair tolerance to one-lung ventilation, surgical trauma, and postoperative rehabilitation. While age is non-modifiable, its identification as a strong independent predictor underscores the imperative for enhanced vigilance and tailored perioperative management in this demographic. Beyond routine care, elderly patients may benefit from a comprehensive geriatric assessment incorporating frailty screening, cognitive evaluation, and nutritional status assessment. Early involvement of geriatric medicine specialists, implementation of delirium prevention protocols, and proactive mobilization strategies could further improve outcomes. Enhanced monitoring in the immediate postoperative period, including more frequent vital sign checks and early warning score tracking, should be considered for this age group.

In addition, preoperative anemia (Hb < 12 g/dL) is an important, potentially modifiable risk factor. Anemia reduces oxygen-carrying capacity, potentially leading to tissue hypoxia, which in turn affects wound healing and increases the risk of infection (20). In lobectomy, maintaining adequate oxygen delivery is crucial due to potential intraoperative blood loss and temporary postoperative impairment of lung function. Anemia can also increase cardiac workload, exacerbate inflammatory responses, and hinder recovery (21, 22). Our results reinforce the general principle of preoperative anemia management and highlight its particular criticality in elderly patients undergoing major thoracic surgery. The use of a 12 g/dL cutoff is consistent with the World Health Organization’s diagnostic criterion for anemia in women and is widely adopted in surgical risk assessment (23). Specific interventions may include oral or intravenous iron supplementation, erythropoiesis-stimulating agents in selected cases, and preoperative transfusion when clinically indicated based on individual patient characteristics and surgical urgency.

Interestingly, despite being well-established risk factors in the general surgical population, COPD and smoking history did not emerge as independent predictors in our multivariable model. Several explanations may account for this finding. First, the study cohort consisted exclusively of elderly patients (≥65 years) who were deemed fit for surgery, potentially representing a selected subgroup with better-than-average physiological reserve among those with COPD or smoking history. This “healthy survivor” effect may have attenuated the impact of these factors on postoperative outcomes. Second, the high prevalence of smoking history in our cohort reduced the contrast between smokers and non-smokers, limiting the statistical power to detect a significant association. Third, the effects of COPD may have been partially captured by other variables in the model, such as ACCI or preoperative pulmonary function tests, which reflect overall respiratory reserve and comorbidity burden. Finally, the relatively modest sample size may have been insufficient to detect small but clinically meaningful independent effects of these factors. These considerations highlight the need for larger, multi-center studies to further elucidate the role of COPD and smoking in elderly patients undergoing pulmonary lobectomy.

From a practical standpoint, this three-point risk score can be easily integrated into routine clinical workflows. In the outpatient setting, surgeons can quickly calculate the score using age, ACCI, and hemoglobin—all routinely available variables. Based on our data, patients with a score of ≥2 face a postoperative major complication rate exceeding 50%, suggesting this threshold might serve as a reasonable trigger for preoperative optimization, including nutritional support, anemia correction, and pulmonary rehabilitation. For those with a score of 3, whose complication risk surpasses 60%, multidisciplinary team involvement and planned intensive care unit admission may be warranted. On the ward, the score could help allocate resources more efficiently—high-risk patients might benefit from closer postoperative monitoring and more aggressive rehabilitation protocols. Admittedly, these proposed thresholds and strategies require prospective validation, but they offer a practical reference point for clinical decision-making in the meantime.

Several limitations of this study should be acknowledged. First, its single-center retrospective design may introduce selection and information biases, limiting the generalizability of the findings. Second, no formal sample size estimation was performed before the study, and the 212 cases included represent a convenience sample from our institution. While this sample size was adequate to identify the main independent predictors in the primary analysis, it limited the statistical power for detailed subgroup analyses (e.g., by specific surgical approach or comorbidity profile) and precluded meaningful interaction analyses to explore effect modification across different patient subgroups. Third, although model diagnostics confirmed adequate calibration (Hosmer-Lemeshow test *p* = 0.562) and no substantial multicollinearity (all VIF < 5), no adjustment was made for multiple comparisons given the exploratory nature of this study; this may increase the risk of type I error and should be considered when interpreting the findings. Fourth, despite adjusting for key confounders, residual confounding may persist due to unmeasured or imperfectly captured variables. For instance, ASA classification can be subject to inter-assessor variability, and differences in surgical technique or perioperative management among multiple operating surgeons could influence outcomes. Finally, the proposed risk score requires external validation in independent, preferably multi-center cohorts to confirm its generalizability, stability, and clinical utility across diverse healthcare settings.

## 5 Conclusion

This study suggests that age ≥70 years, ACCI score ≥6, and preoperative anemia are independent predictors of major complications after lobectomy in elderly patients. Risk stratification based on these three indicators can provide a reference for the rational allocation of resources for preoperative communication, targeted pre-rehabilitation (such as correction of anemia), and postoperative monitoring. Future prospective studies with larger samples and more comprehensive designs, especially in a multicenter setting, are needed to further validate the reliability of these findings and explore the actual effects of systematic interventions based on this risk stratification on improving patient outcomes.

## Data Availability

The original contributions presented in the study are included in the article/Supplementary material, further inquiries can be directed to the corresponding author.
