# Supplementary material for: Predictors of postoperative complications in elderly patients undergoing pulmonary lobectomy: a retrospective cohort study
Source: Front Med (Lausanne). 2026 Aug 11;13:1789608. doi: 10.3389/fmed.2026.1789608 (PMC13503564; doi:10.3389/fmed.2026.1789608)
Supplement: Supplementary file 1 [file Table_1.DOCX]

**Supplementary Table S1. Summary of the Age-Adjusted Charlson Comorbidity Index (ACCI) framework.**

| **Component** | **Definition / examples** | **Weight** |
| --- | --- | --- |
| Myocardial infarction | History of myocardial infarction | 1 |
| Congestive heart failure | Chronic or prior heart failure | 1 |
| Peripheral vascular disease | Peripheral arterial disease, aneurysm, etc. | 1 |
| Cerebrovascular disease | Stroke or transient ischemic attack | 1 |
| Dementia | Any documented dementia syndrome | 1 |
| Chronic pulmonary disease | Chronic obstructive pulmonary disease or other chronic pulmonary disorders | 1 |
| Connective tissue disease | Rheumatologic/connective tissue disorders | 1 |
| Peptic ulcer disease | Documented peptic ulcer disease | 1 |
| Mild liver disease | Chronic hepatitis or mild chronic liver disease without major complications | 1 |
| Diabetes without end-organ damage | Diabetes mellitus without documented microvascular or macrovascular end-organ damage | 1 |
| Hemiplegia/paraplegia | Persistent major motor deficit | 2 |
| Moderate or severe renal disease | Chronic kidney disease with significant renal impairment | 2 |
| Diabetes with end-organ damage | Diabetes with nephropathy, retinopathy, neuropathy, or other organ damage | 2 |
| Any non-metastatic solid tumor | Solid malignancy without distant metastasis* | 2 |
| Leukemia | Any leukemia | 2 |
| Lymphoma | Any lymphoma | 2 |
| Moderate or severe liver disease | Cirrhosis or liver disease with major complications | 3 |
| Metastatic solid tumor | Solid malignancy with distant metastasis | 6 |
| AIDS/HIV | Acquired immunodeficiency syndrome / HIV infection | 6 |
| **Age adjustment** | 50–59 years | **+1** |
|  | 60–69 years | **+2** |
|  | 70–79 years | **+3** |
|  | ≥80 years | **+4** |

**Abbreviations:** ACCI, Age-Adjusted Charlson Comorbidity Index; HIV, human immunodeficiency virus; AIDS, acquired immunodeficiency syndrome.

**Supplementary Table S2. Summary of the Clavien–Dindo classification system used for postoperative complication grading.**

| **Grade** | **Definition** | **Practical interpretation** |
| --- | --- | --- |
| Grade I | Any deviation from the normal postoperative course without the need for pharmacological treatment or surgical, endoscopic, or radiological intervention | Minor complication requiring only basic bedside measures |
| Grade II | Complication requiring pharmacological treatment beyond that allowed for grade I | Complication requiring drug treatment, such as antibiotics, blood transfusion, or total parenteral nutrition |
| Grade IIIa | Complication requiring surgical, endoscopic, or radiological intervention **not** under general anesthesia | Intervention required, but no general anesthesia |
| Grade IIIb | Complication requiring surgical, endoscopic, or radiological intervention **under** general anesthesia | Invasive intervention under general anesthesia |
| Grade IVa | Life-threatening complication requiring ICU management, with **single-organ dysfunction** | Severe complication requiring intensive care support |
| Grade IVb | Life-threatening complication requiring ICU management, with **multi-organ dysfunction** | Critical complication with multiple organ failure |
| Grade V | Death of the patient | Postoperative mortality |

**Abbreviations:** ICU, intensive care unit.
